# Impact of cyclodextrins on the behavior of amphiphilic ligands in aqueous organometallic catalysis

**DOI:** 10.3762/bjoc.8.167

**Published:** 2012-09-06

**Authors:** Hervé Bricout, Estelle Léonard, Christophe Len, David Landy, Frédéric Hapiot, Eric Monflier

**Affiliations:** 1 Université Lille Nord de France, CNRS UMR 8181, Unité de Catalyse et de Chimie du Solide - UCCS, UArtois, Faculté des Sciences Jean Perrin, SP18, 62307 Lens Cedex, France; 2 Université de Technologie de Compiègne, Transformation Intégrée de la Matière Renouvelable, EA 4297 UTC/ESCOM Centre de recherches de Royallieu, BP 20529, F-60205 Compiègne Cedex, France; 3 Université Lille Nord de France, UCEIV, ULCO, 145, Avenue Maurice Schumann, MREI 1, F-59140 Dunkerque, France

**Keywords:** amphiphilic phosphanes, biphasic system, cyclodextrins, micelles

## Abstract

In this study, we showed that the addition of randomly modified β-cyclodextrin (RAME-β-CD) in aqueous medium could have a beneficial impact on the catalytic performances of phosphane-based aggregates in the Pd-catalyzed cleavage of allyl carbonates (Tsuji–Trost reaction). The RAME-β-CD/phosphane supramolecular interactions helped explain the catalytic results. The presence of RAME-β-CD in the aqueous compartment improved the phosphane-based aggregate dynamics. The exchanges between the hydrophobic substrate-containing aggregate core and the catalyst-containing aqueous phase were then greatly favored, resulting in an increase in the catalytic performances.

## Introduction

Facing the need of developing a greener chemistry, chemists have recently focused their investigations on clean transformation processes to convert organic molecules into products. Traditional solvents have especially been incriminated due to the pollution problems they raise. Thus, in the context of increased awareness about environmental safety, nonconventional media appeared to be a promising alternative to toxic solvents. The use of supercritical fluids, ionic liquids and water especially appeared to be an effective solution to limit the environmental impact of chemical reactions [1–3]. Although these media all display eco-friendly characteristics such as non-flammability and chemical stability, water has an additional advantage of being cheap, nontoxic and available in large amounts. As an example, the concept of aqueous-phase organometallic catalysis implemented by E. Kuntz in the 70s clearly illustrates the potential of water as a reaction medium [4–5]. However, while hydrophilic substrates can be readily converted in water, the conversion of hydrophobic substrates constitutes a real challenge not completely solved so far. Recently, we showed that an association of cyclodextrins (CDs) and amphiphilic phosphanes could significantly improve the catalytic performances in an aqueous rhodium-catalyzed hydroformylation of higher olefins [6]. Herein we clearly demonstrate that the beneficial effect of CDs on a catalytic micellar system can be generalized to another reaction and other amphiphilic phosphanes. To this end, we undertook a study using the randomly methylated β-cyclodextrin (RAME-β-CD) as an additive in an aqueous Pd-catalyzed cleavage reaction of allyl carbonates (Tsuji–Trost reaction) and four amphiphilic phosphanes as aggregate-building blocks. The RAME-β-CD/phosphane interaction and its consequence on the catalytic results are discussed.

## Results and Discussion

**Figure 1:**
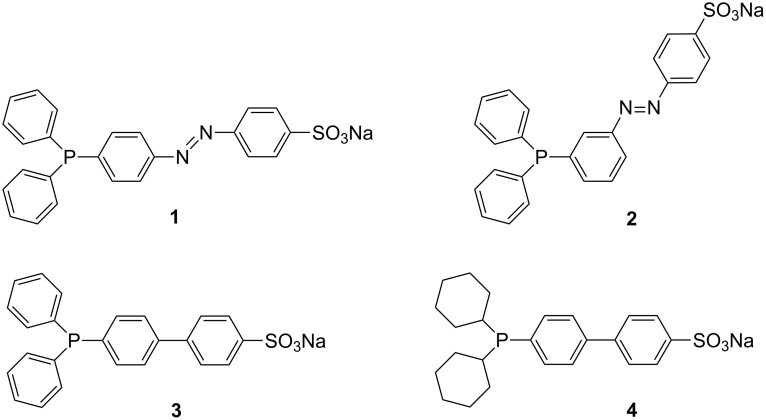
Water-soluble phosphanes **1**–**4**.

To expand the scope of the CD/amphiphilic phosphane combination in aqueous-phase organometallic catalysis, four amphiphilic phosphanes **1**–**4** (Figure 1) were considered [6–8].

These phosphanes have been chosen for the information they could give under catalytic conditions by considering their structures and their amphiphilic properties. In this study, each phosphane has been proven to supramolecularly interact with β-CDs. For example, addition of a stoichiometric amount of RAME-β-CD on aqueous solutions of water-soluble phosphanes **1**–**4** led to the formation of phosphane
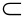
RAME-β-CD supramolecular complexes. The stoichiometry and association constant *K*_ass_ of these complexes were measured by isothermal titration calorimetry (ITC), at a suitable RAME-β-CD/phosphane concentration ratio ensuring the absence of micelles during the whole experiment. Under these conditions, the values reflect the interaction between a single CD with a single phosphane only. A 1/1 stoichiometry was found whatever the phosphane, according to the fit between theoretical and experimental heats. The *K*_ass_ values were very dependent upon the phosphane structure. The *K*_ass_ values measured for **1** and **2** (300 and 590 M^−1^, respectively) were three orders of magnitude lower than those measured for **3** and **4** (326 000 and 210 000 M^−1^, respectively) indicative of the deleterious role played by the diazo function on the inclusion phenomenon. In fact, two distinct parameters could be implicated to explain the low *K*_ass_ values observed for **1** and **2**. First, the hydrophilic diazo function had less affinity for the hydrophobic CD cavity than the biphenyl moiety. Additionally, the phosphane nonlinear geometry resulting from the zig-zag diazo structure also disfavored the recognition process between the phosphane and the CD cavity.

**Figure 2:**
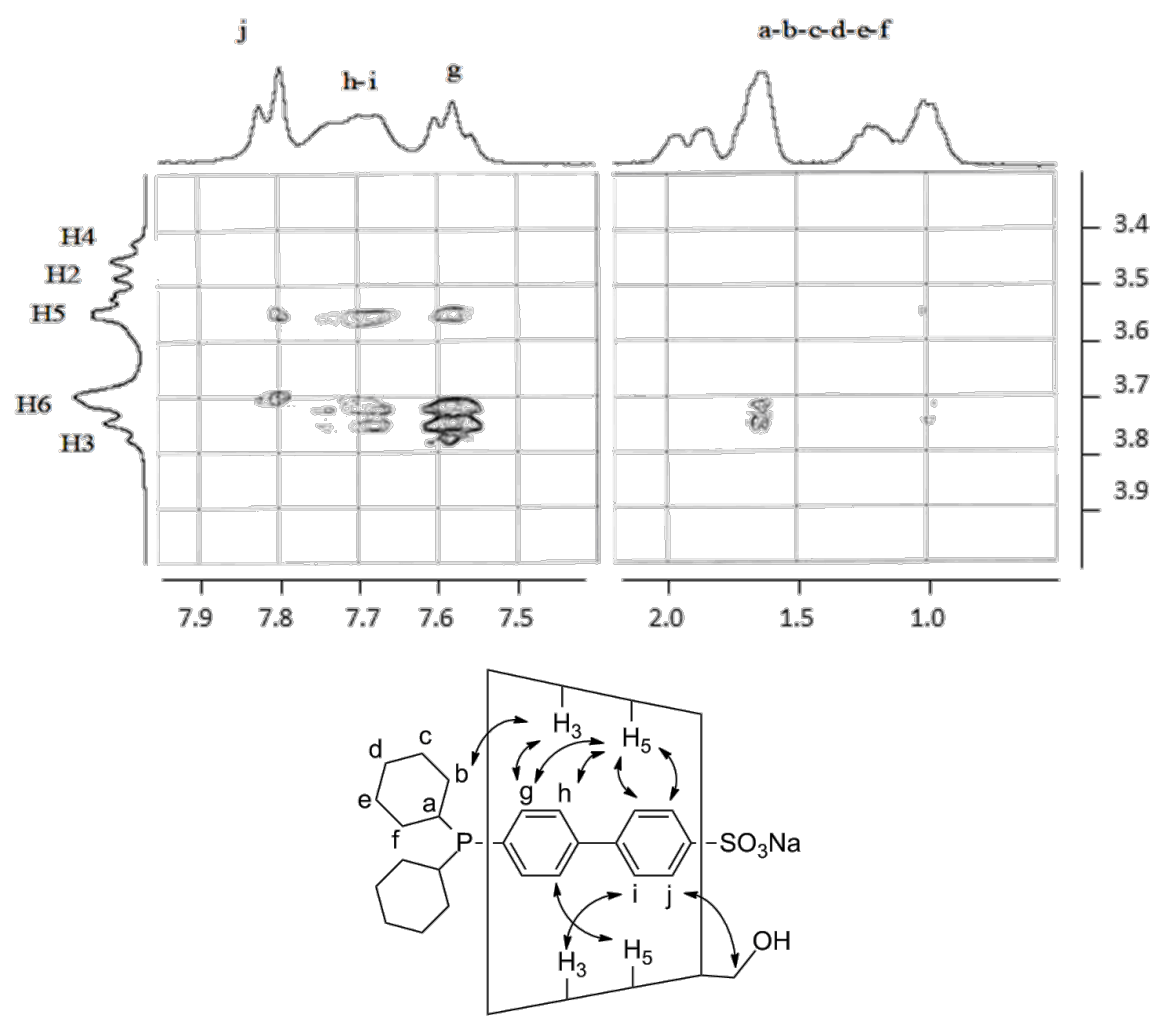
2D T-ROESY NMR spectrum of a stoichiometric mixture of β-CD and **4** (3 mM each) in D_2_O at 20 °C.

The existence of phosphane
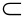
CD supramolecular complexes was confirmed by using a 2D NMR T-ROESY sequence sensitive to dipolar contacts between the CD host and the phosphane guest. Correlations were detected on T-ROESY spectra of stoichiometric mixtures of RAME-β-CD and phosphanes **1**–**4**. However, a clear assignment of the CD and phosphane protons could not be properly made due to a severe broadening of the NMR signals (ESI). Accordingly, the native β-CD was chosen as host by default. The CD protons could then be assigned as illustrated in Figure 2 for the **4**
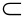
β-CD supramolecular complex.

Cross peaks were detected between the inner CD protons H-3/H-5 and the phosphane aromatic protons. A correlation was also detected between some of the cyclohexyl protons and H-3 suggesting a deep phosphane inclusion into the CD cavity. For the other phosphanes, mixtures of supramolecular complexes could be detected because of the additional inclusion of the phosphorus-bound phenyl groups into the CD cavities (ESI). It should be noted that the signal broadening observed for phosphane protons suggested the presence of aggregates in the medium. Thus, though a 1/1 CD/phosphane mixture was used, phosphane-based aggregates and native β-CD/phosphane complexes coexisted in water in this concentration range [9]. In fact, the mixture of CD/phosphane complexes and aggregates was a consequence of the competitive equilibria of CD/phosphane complexation and phosphane self-assembly to form micelles.

**Figure 3:**
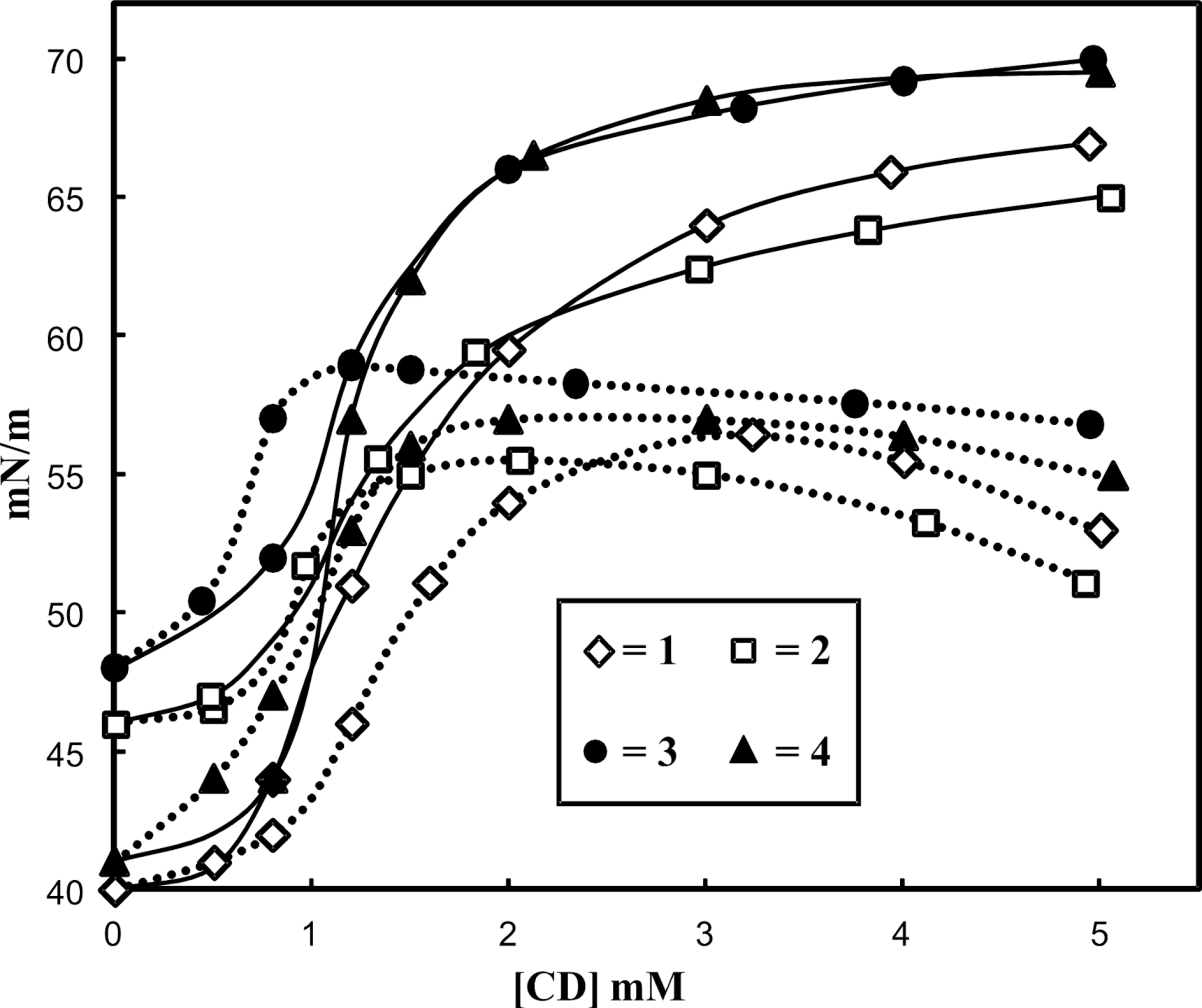
Effect of increasing concentrations of β-CD (solid lines) and RAME-β-CD (dotted lines) on the surface tension of 1 mM aqueous solutions of phosphanes **1**–**4** (20 °C).

**Figure 4:**
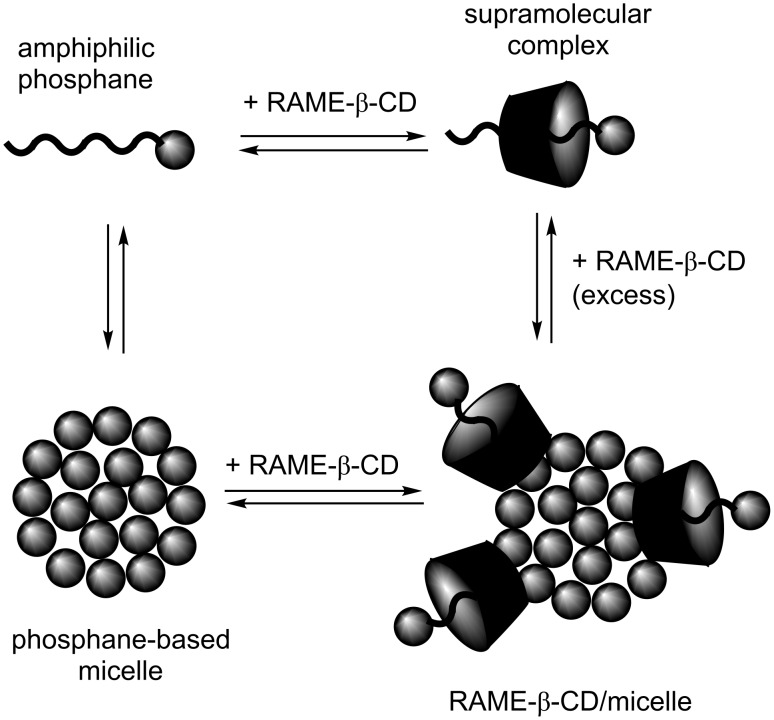
Equilibria in a phosphane-based micelle/RAME-β-CD mixture.

The surface activity of the four studied phosphanes has already been the subject of investigations [7–8]. Phosphanes **1**–**4** proved to be very surface active as expected from their amphiphilic structure (ESI). However, we show in this study that the addition of increasing amounts of native β-CD or RAME-β-CD in phosphane-containing solutions produced a significant rise of the surface tension (Figure 3). This phenomenon was interpreted in terms of CD/amphiphilic phosphane interaction leading, for high CD concentrations, to an aggregate-destructuring process. Indeed, in the post-micellar region, increasing the CD amount resulted in a displacement of the equilibria towards the CD/phosphane complex. The aggregates were then partially or totally destroyed depending on the CD concentration. Mixtures of modified and unaltered phosphane-based aggregates probably coexisted in water (Figure 4). The above results corroborate previous studies on CD/amphiphilic phosphane interactions [9–13].

**Scheme 1:**
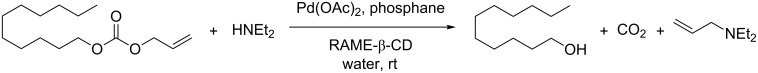
Tsuji–Trost reaction mediated by a phosphane-based micelle/RAME-β-CD combination.

The catalytic performances of the CD/amphiphilic phosphane combination have been evaluated in the aqueous Pd-catalyzed cleavage reaction of allyl undecyl carbonate (Scheme 1).

**Figure 5:**
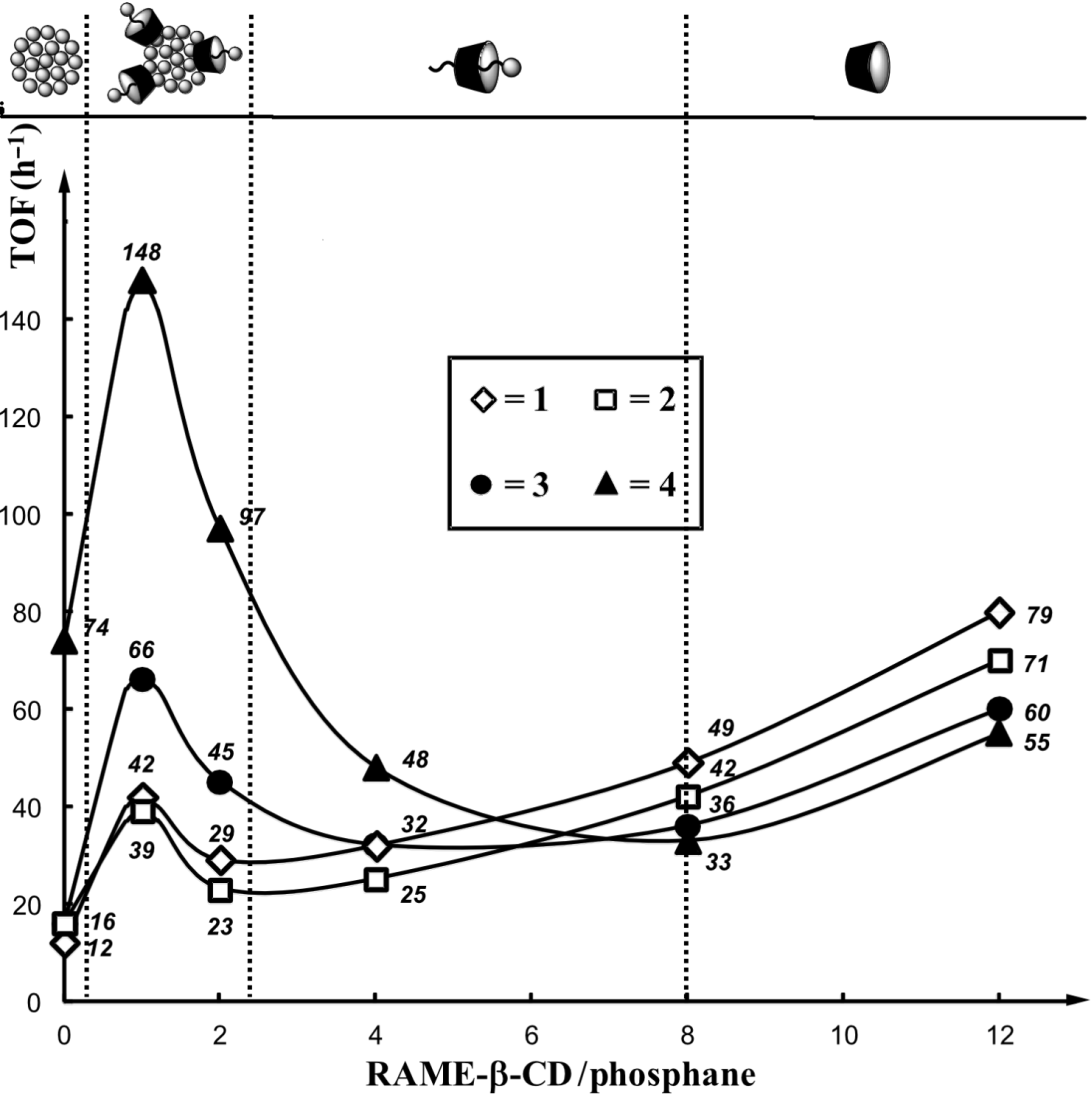
Turnover frequency (TOF) as a function of the RAME-β-CD/phosphane ratio in the Pd-catalyzed cleavage of allyl undecyl carbonate. Reaction conditions: Pd(OAc)_2_ = 2.23 μmol, phosphane = 20.1 μmol, RAME-β-CD = 0–241 μmol, H_2_O = 2 g, allyl undecyl carbonate = 223 μmol, diethylamine = 446 μmol, dodecane = 110 μmol, heptane = 2 g, 1250 rpm, room temperature. The TOF was defined as the number of moles of allyl undecyl carbonate converted per mole of palladium and per hour in the early stage of the reaction (20–40% of conversion).

The reactions were performed at room temperature under nitrogen by using palladium acetate as a catalyst precursor, phosphanes **1**–**4** as hydrosoluble ligands, and diethylamine as an allyl scavenger. RAME-β-CD was chosen as an additive because of its ability to adsorb at the aqueous/organic interface [14–17]. The RAME-β-CD/phosphane ratio was varied to evaluate the impact of the CD concentration on the catalytic micellar system. The catalytic results have been translated in terms of turnover frequency (TOF) to RAME-β-CD/phosphane ratio, thus highlighting the role of RAME-β-CD on the catalytic performances of the phosphane-based aggregates (Figure 5).

Nonlinear curves were obtained indicative of different processes depending on the CD concentration. The highest TOFs were measured for a RAME-β-CD/phosphane ratio of 1:1, while a huge drop in TOF was observed for a RAME-β-CD/phosphane ratio of 2:1. Conversely, high RAME-β-CD/phosphane ratios yielded an increase in TOF. The results could be interpreted as follows. First, for a RAME-β-CD/phosphane ratio of around 1:1, the micelle structure was slightly altered because of the interaction of RAME-β-CD with the micelle constituents, especially the inclusion process (Figure 4).

As the micelle dynamics was improved, the exchanges between the hydrophobic substrate-containing micelle core and the catalyst-containing aqueous solution were then greatly favored, thus leading to an increase in TOF. In that case, the substrate concentration within the core of the phosphane-based aggregates remained high. Note that the beneficial effect of RAME-β-CD on the aggregates was observed in a well-defined concentration range for each phosphane. The concentration range was narrow for aggregates constituted by diazo phosphanes **1** or **2** and rather broad for aggregates constituted by **3** or **4**. Second, above a stoichiometric RAME-β-CD/phosphane ratio, the RAME-β-CD impact on aggregates became deleterious to the point that the aggregates were destroyed. Most of the RAME-β-CD cavities were then filled with phosphanes and the equilibria were greatly displaced towards the RAME-β-CD/phosphane complex. In that case, neither the amphiphilic phosphanes nor RAME-β-CD could favor the substrate–catalyst binding anymore. On the contrary, their interaction was counterproductive as both components annihilated their interfacial properties. The observed TOFs measured in this concentration range probably resulted from the intrinsic catalyst activity. Finally, the TOF variations observed for very high RAME-β-CD/phosphane ratios could be easily rationalized considering the ability of RAME-β-CD to supramolecularly recognize not only the phosphane but also the substrate. When mixed in excess with amphiphilic phosphanes, a proportion of the RAME-β-CD included phosphanes in their cavities, but the majority of RAME-β-CD interacted with allyl carbonates and acted as mass transfer promoters between the substrate-containing organic phase and the aqueous component [10–13].

It should be noted that the above catalytic performances were mainly related to the *K*_ass_ values. Indeed, the spread between the highest and the lowest TOF was larger for phosphanes **3** and **4** for which *K*_ass_ values over 200 000 M^−1^ were measured. With the RAME-β-CD/interaction being strong, the aggregate dynamics was greatly favored at a CD/phosphane ratio of around 1:1 (high TOF). In that case, the intensity of the aggregate destructuring process was stronger at a higher CD/phosphane ratio (low TOF) as a result of the formation of stable RAME-β-CD/phosphane complexes. Conversely, because of the low *K*_ass_ values measured for phosphanes **1** and **2**, the amplitude between the highest and the lowest TOF was moderate, illustrative of the weak interaction between both components. However, for stoichiometric RAME-β-CD/phosphane mixtures (10 mM each, catalytic conditions), the percentages of RAME-β-CD/phosphane complexes were calculated to be 56.6, 66.4, 98.3 and 97.8% for phosphanes **1**, **2**, **3** and **4**, respectively. Thus, even for phosphanes **1** and **2** whose *K*_ass_ were low, a significant amount of RAME-β-CD/phosphane complexes were present in the aqueous medium and significantly contribute to the beneficial effect observed for stoichiometric RAME-β-CD/phosphane mixtures.

Accordingly, in this study we showed that RAME-β-CD could positively interact with phosphane-containing aggregates in a very narrow concentration range to improve the exchanges between the hydrophobic aggregate core and the aqueous catalyst-containing solution. The result is in line with the conclusions previously drawn by using other modified β-CDs as additives [6]. The beneficial effect of the CD/amphiphilic phosphane interaction thus appeared generalizable to other phosphanes and other reactions and to reinforce the view of a synergetic relationship between both components.

## Conclusion

In our constant effort to elaborate an effective catalytic system aiming at converting hydrophobic substrates in water, the CD/amphiphilic phosphane combination proved to be a versatile solution provided that the ratio between both components had been carefully defined. The CD positively affects the phosphane-constituted aggregate structure by supramolecular means to the point that the substrate–catalyst binding becomes favorable. Given the above catalytic results, we are currently widening the scope of the CD/amphiphilic phosphane interaction in other aqueous-phase organometallic reactions.

## Supporting Information

File 1 Experimental procedures and characterization of the supramolecular complexes.
