# Supplementary material for: Impact of cyclodextrins on the behavior of amphiphilic ligands in aqueous organometallic catalysis
Source: Beilstein J Org Chem. 2012 Sep 6;8:1479–84. doi: 10.3762/bjoc.8.167 (PMC3458772; doi:10.3762/bjoc.8.167)
Supplement: File 1 — Experimental procedures and characterization of the supramolecular complexes. [file Beilstein_J_Org_Chem-08-1479-s001.pdf]

Supporting Information

for

Impact of cyclodextrins on the behavior of amphiphilic ligands in aqueous organometallic catalysis

Hervé Bricout[1], Estelle Léonard[2], Christophe Len[2], David Landy[3], Frédéric Hapiot*[1], and Eric Monflier*[1]

Address:

[1]Université Lille Nord de France, CNRS UMR 8181, Unité de Catalyse et de Chimie du Solide - UCCS, UArtois, Faculté des Sciences Jean Perrin, SP18, 62307 Lens Cedex, France, [2]Université de Technologie de Compiègne, Transformation Intégrée de la Matière Renouvelable, EA 4297 UTC/ESCOM Centre de recherches de Royallieu, BP 20529, F-60205 Compiègne Cedex, France and [3]Université Lille Nord de France, UCEIV, ULCO, 145, Avenue Maurice Schumann, MREI 1, F-59140 Dunkerque, France

E-mail: Eric Monflier* - eric.monflier@univ-artois.fr;

Frédéric Hapiot - frederic.hapiot@univ-artois.fr

* Corresponding author

Experimental procedures and characterization of the supramolecular complexes

CONTENTS

	page
Materials - analysis	S3
Surface tension measurements	S5
ITC measurements	S6
2D T-ROESY spectra	S10
Palladium-catalyzed Tsuji–Trost reaction	S16

Materials

All chemicals were purchased from Strem Chemicals and Aldrich Chemicals in their highest purity. Distilled water was used in all experiments. All solvents and liquid reagents were degassed by bubbling nitrogen for 15 min before each use or by two freeze–pump–thaw cycles.

Analysis

NMR spectroscopy

NMR spectra were recorded on a Bruker DRX300 spectrometer operating at 300 MHz or 500 MHz for ^1H nuclei. $CDCl_3$ (99.50% isotopic purity), DMSO-d_6 (99.80% isotopic purity) and D_2O (99.92% isotopic purity) was purchased from Euriso–Top. The 2D T-ROESY experiments were run using the software supplied by Bruker. T-ROESY experiments were preferred to classical ROESY experiments as this sequence provides reliable dipolar cross-peaks with a minimal contribution of scalar transfer. Mixing times for T-ROESY experiments were set at 300 ms. The data matrix for the T-ROESY was made of 512 free induction decays, 1 K points each, resulting from the co-addition of 32 scans. The real resolution was 1.5–6.0 Hz/point in F2 and F1 dimensions, respectively. They were transformed in the nonphase-sensitive mode after QSINE window processing. Note that high concentrations were used (3 mM each) to overcome detection problems inherent to the 2D T-ROESY NMR sequence.

Isothermal titration calorimetry (ITC)

A ITC_{200} isothermal calorimeter (MicroCal Inc., USA) was used for the calorimetric characterization of the RAME-β-CD/phosphane complex. The release protocol was used [1-3]: A degassed aqueous solution (204.5 µL; phosphate buffer: pH 6.5) was titrated with a degassed solution (same buffer) containing both phosphane and RAMEB in a 40 µL syringe. As the complex was formed within the syringe, each injection led to a partial dissociation of the inclusion compound, as a consequence of dilution within the cell. Within the syringe, concentrations of phosphanes **1–4** were 0.5 mM, while RAME-β-CD was set to 10 mM with phosphanes **1** and **2** and to 0.5 mM with phosphanes **3** and **4**. Owing to the stability of each complex and to dilution

during the experiments, these conditions implied that the free phosphane concentrations never exceeded 0.05 mM in the cell, thus minimizing the existence of micelles and corresponding heat variations. After addition of an initial aliquot of 0.4 μL, 10 aliquots of 3.7 μL of the RAME-β-CD/phosphane solution were delivered (over 7.4 s for each injection). The time interval between two consecutive injections was 150 s and the agitation speed was 1000 rpm for all experiments. For each titration, the heat flow was recorded as a function of time. The areas under the peak following each injection were obtained by integration of the raw signal using the Origin 7.0 software provided by MicroCal. In addition, the heat effects due to dilution of RAME-β-CD were corrected for by performing blank titrations. Binding constant and enthalpy were then determined by nonlinear regression analysis of the release isotherms (assuming a 1:1 equilibrium), according to reference [3], by implementing the corresponding equations in Excel software. It has to be pointed out that even if dissociation is observed in this kind of experiment, analysis of the corresponding data is realized in terms of the formation constant. As a consequence, the theoretical heats were fitted to the experimental ones by varying the formation constant and enthalpy values (χ2 minimisation process, with Newton Raphson algorithm). Each titration experiment was performed three times (at 298 K) to ensure reproducibility of the results.

Gas chromatography

Gas chromatographic analyses were carried out on a Shimadzu GC-17A gas chromatograph equipped with a polydimethylsiloxane capillary column (25 m × 0.25 mm; film thickness: 0.25 μm) and a flame ionization detector (GC:FID).

1. Heerklotz, H. H.; Binder, H.; Epand, R. M. *Biophys. J.* **1999**, *76*, 2606–2613.

2. Illapakurthy, A. C.; Wyandt, C. M.; Stodghill, S. P. *Eur. J. Pharm. Biopharm.* **2005**, *59*, 325–332.

3. Mallard, I.; Landy, D.; Bouchemal, N.; Fourmentin, S. *Carbohydr. Res.* **2011**, *346*, 35-42.

Surface tension measurements

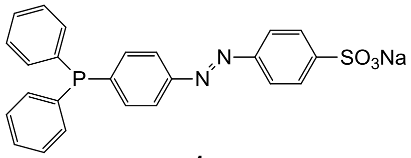

1

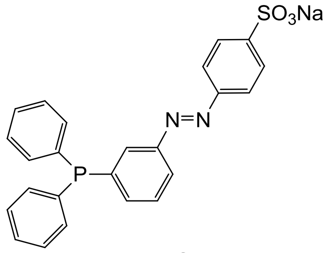

2

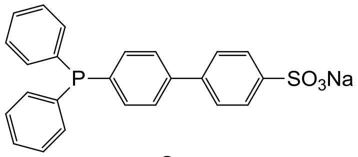

3

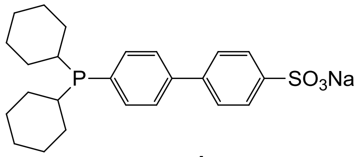

4

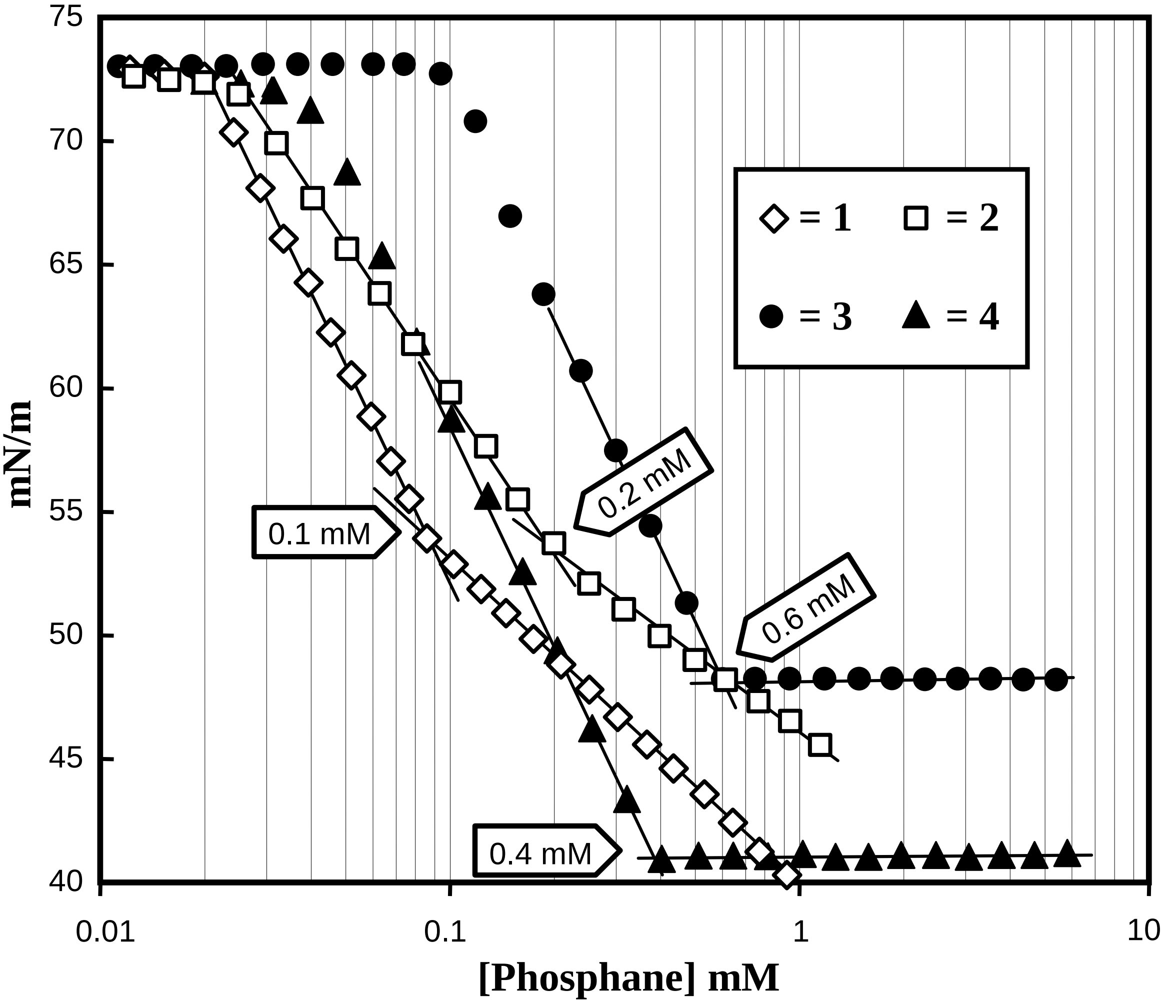

Figure S1: Surface tension of phosphanes **1–4** in water at 20 °C.

ITC measurements

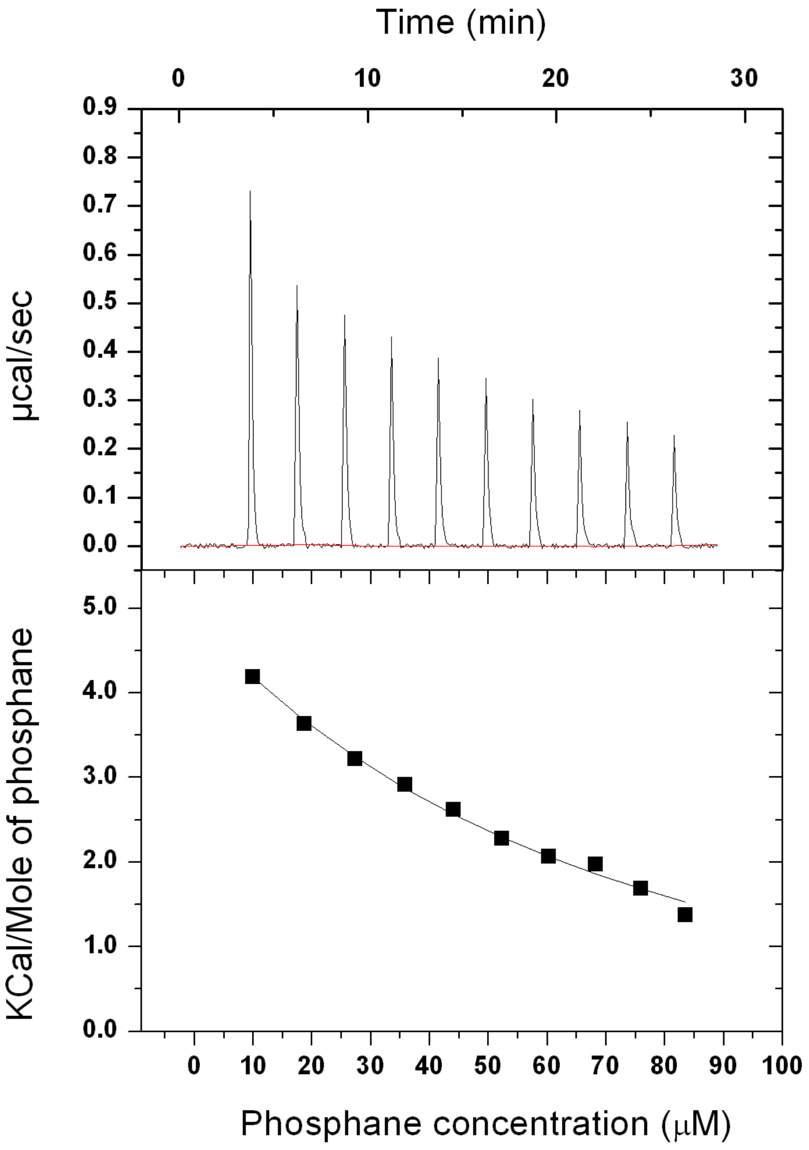

Figure S2: Representative thermogram (upper part) and corresponding binding isotherm (lower part) for ITC release experiments between RAME-β-CD (10 mM) and phosphane **1** (0.5 mM). The solid line represents the best theoretical fit to the experimental points.

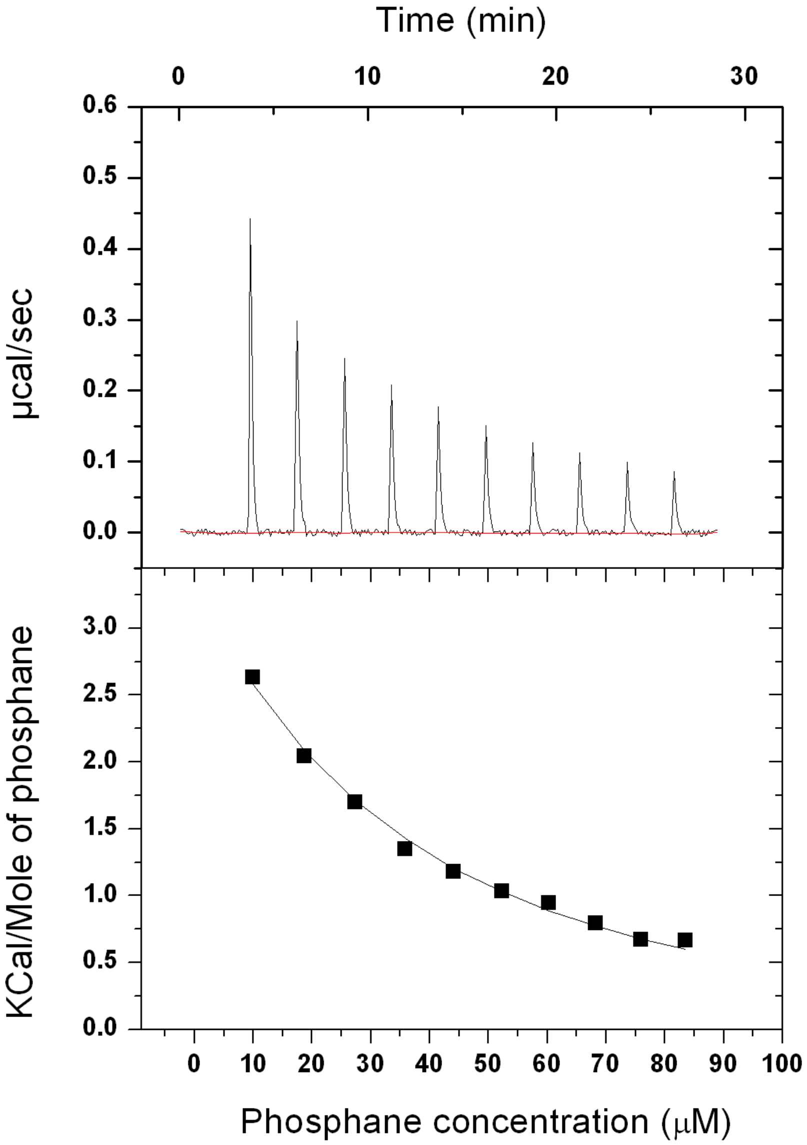

Figure S3: Representative thermogram (upper part) and corresponding binding isotherm (lower part) for ITC release experiments between RAME-β-CD (10 mM) and phosphane **2** (0.5 mM). The solid line represents the best theoretical fit to the experimental points.

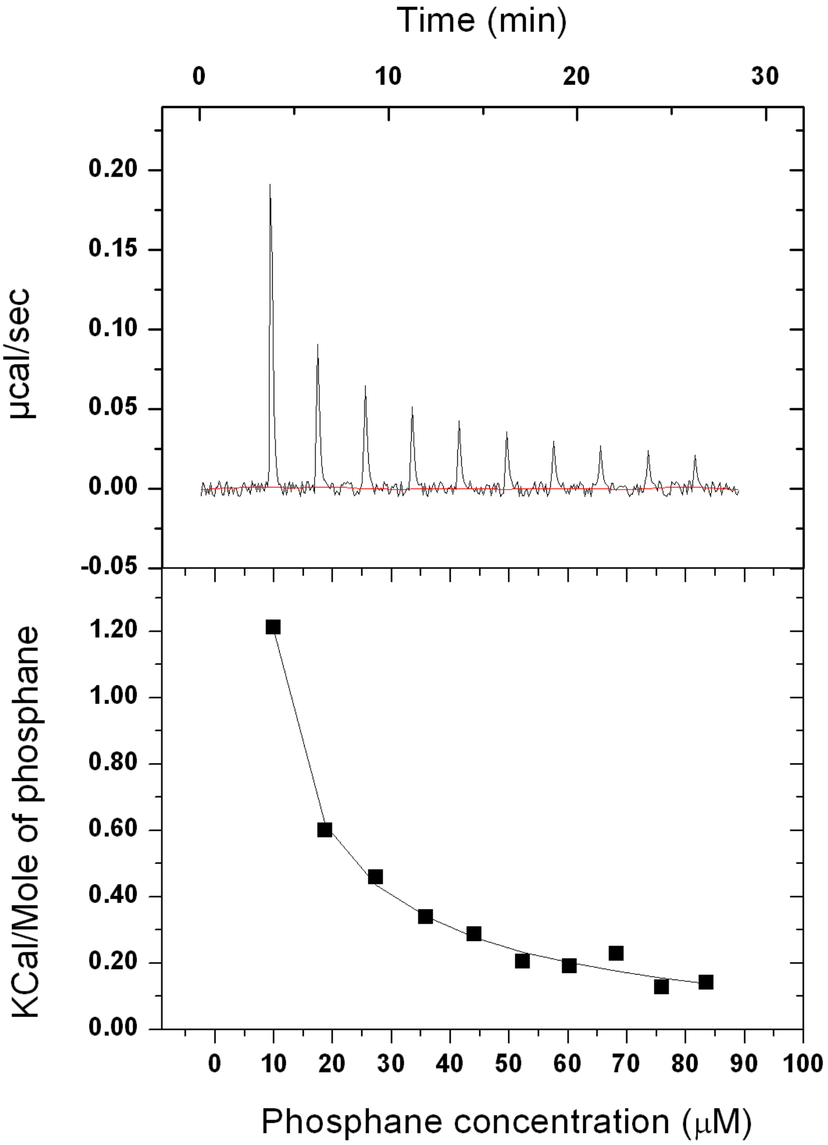

Figure S4: Representative thermogram (upper part) and corresponding binding isotherm (lower part) for ITC release experiments between RAME-β-CD (0.5 mM) and phosphane **3** (0.5 mM). The solid line represents the best theoretical fit to the experimental points.

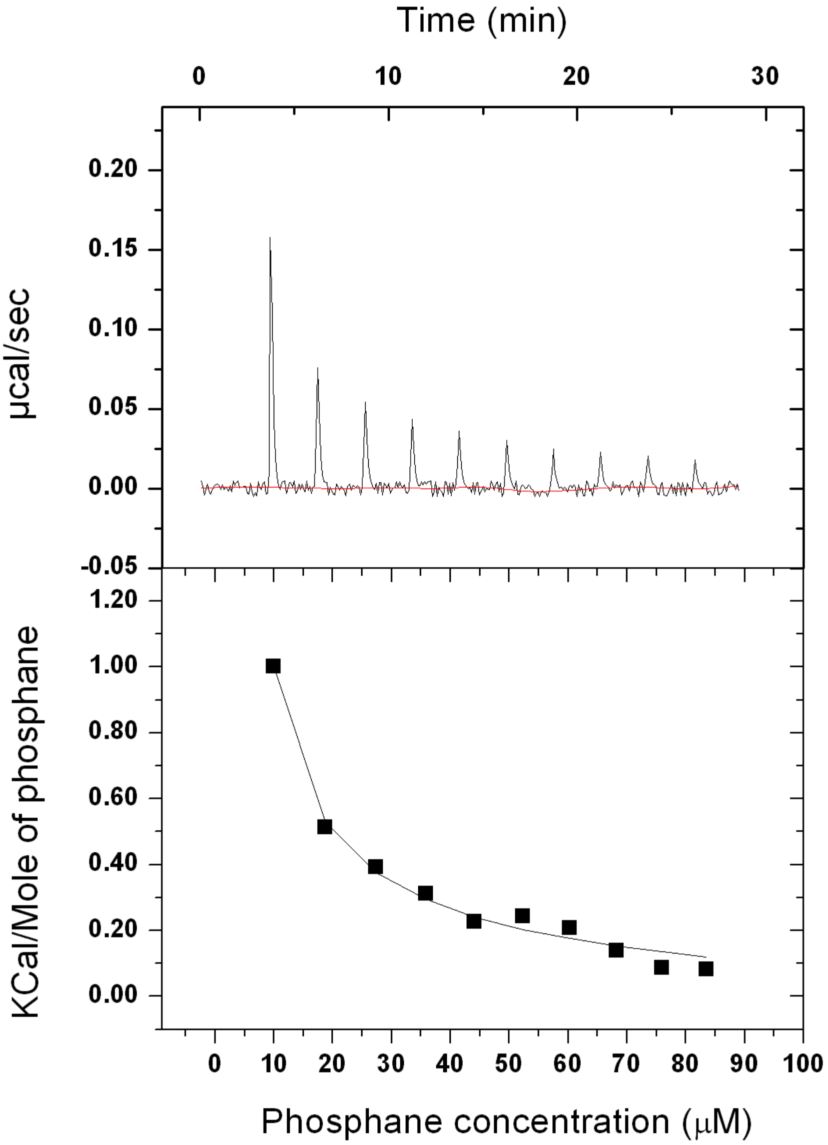

Figure S5: Representative thermogram (upper part) and corresponding binding isotherm (lower part) for ITC release experiments between RAME-β-CD (0.5 mM) and phosphane **4** (0.5 mM). The solid line represents the best theoretical fit to the experimental points.

2D T-ROESY spectra

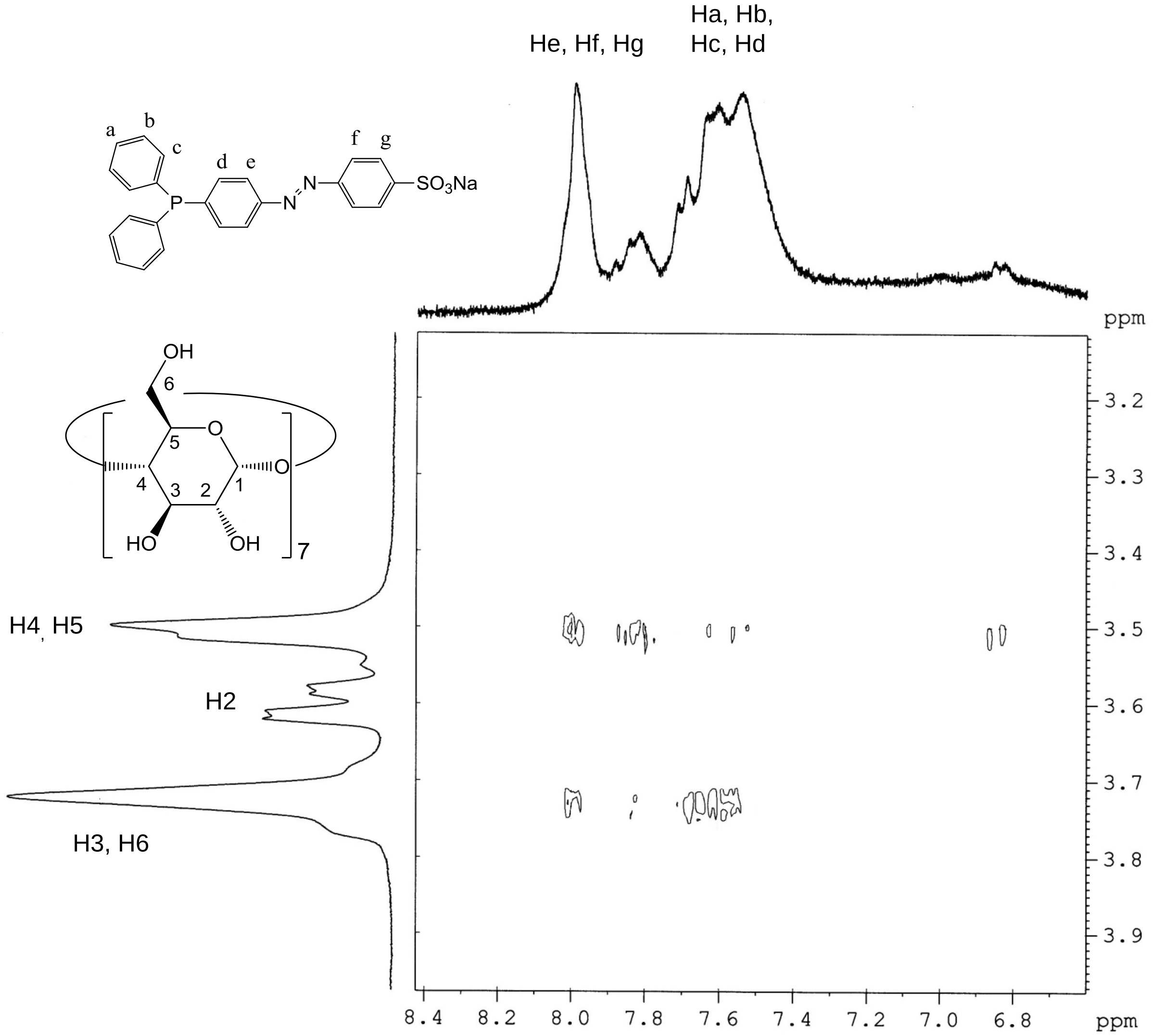

Figure S6: T-ROESY spectrum (300 MHz) of a stoichiometric mixture of native β-CD and phosphane **1** (3 mM each) in D_2O at 20 °C.

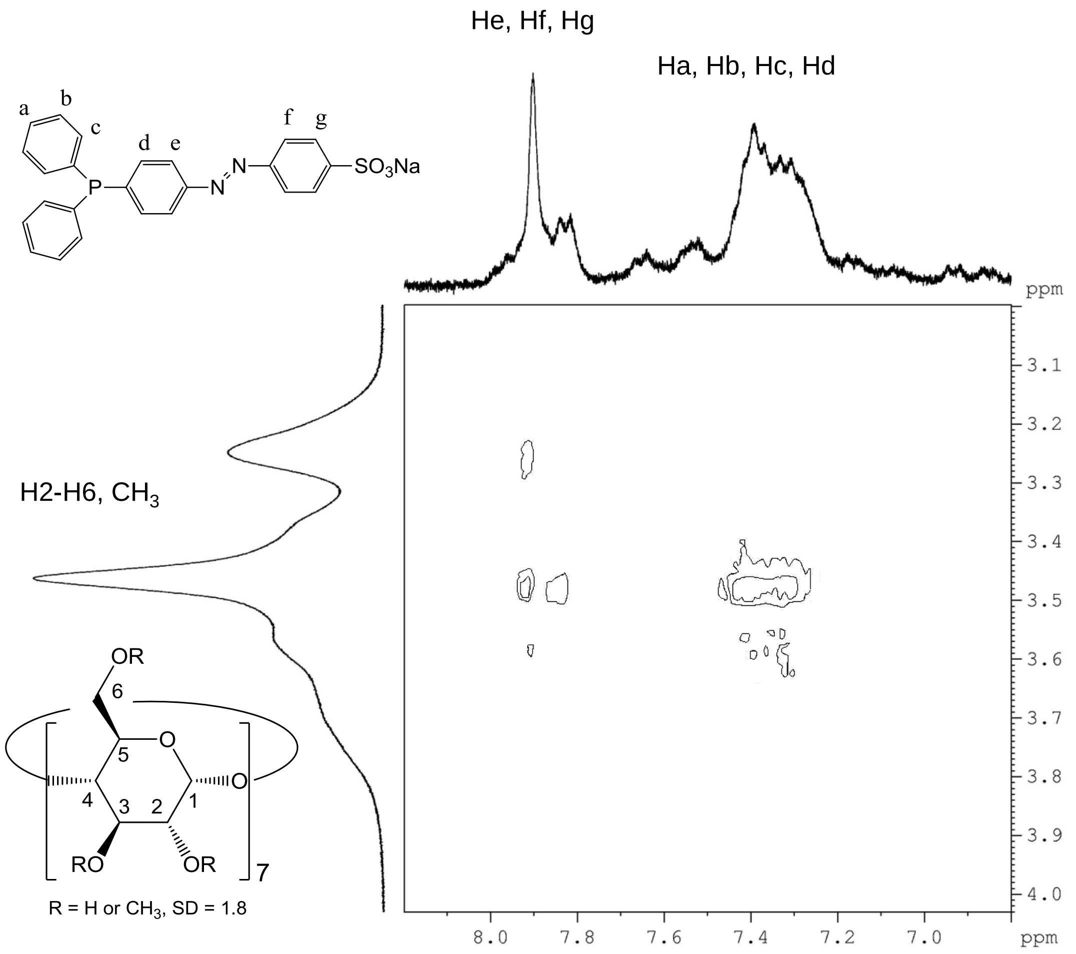

Figure S7: T-ROESY spectrum (300 MHz) of a stoichiometric mixture of RAME-β-CD and phosphane **1** (3 mM each) in D_2O at 20 °C.

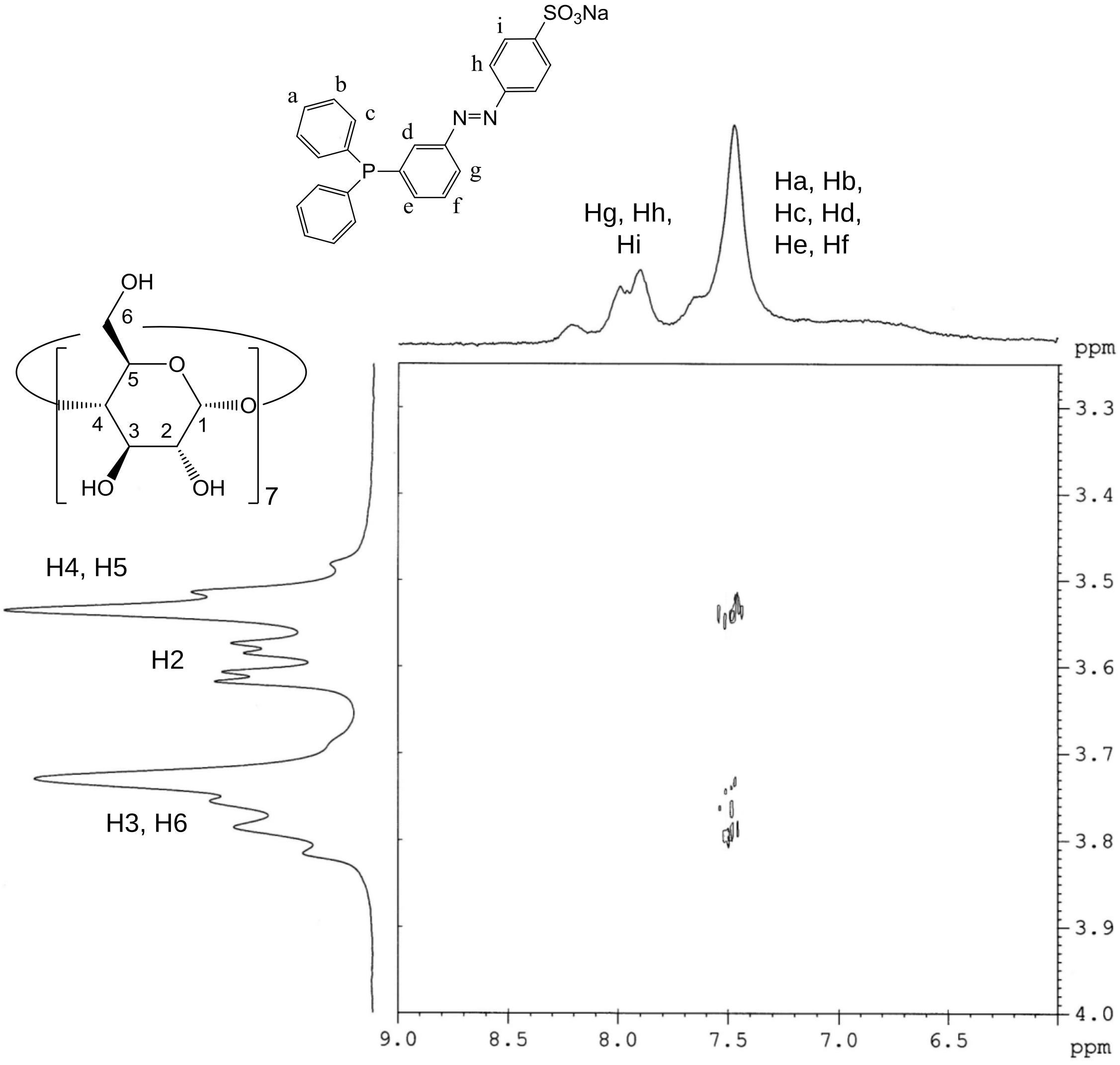

Figure S8: T-ROESY spectrum (300 MHz) of a stoichiometric mixture of native β-CD and phosphane **2** (3 mM each) in D_2O at 20 °C.

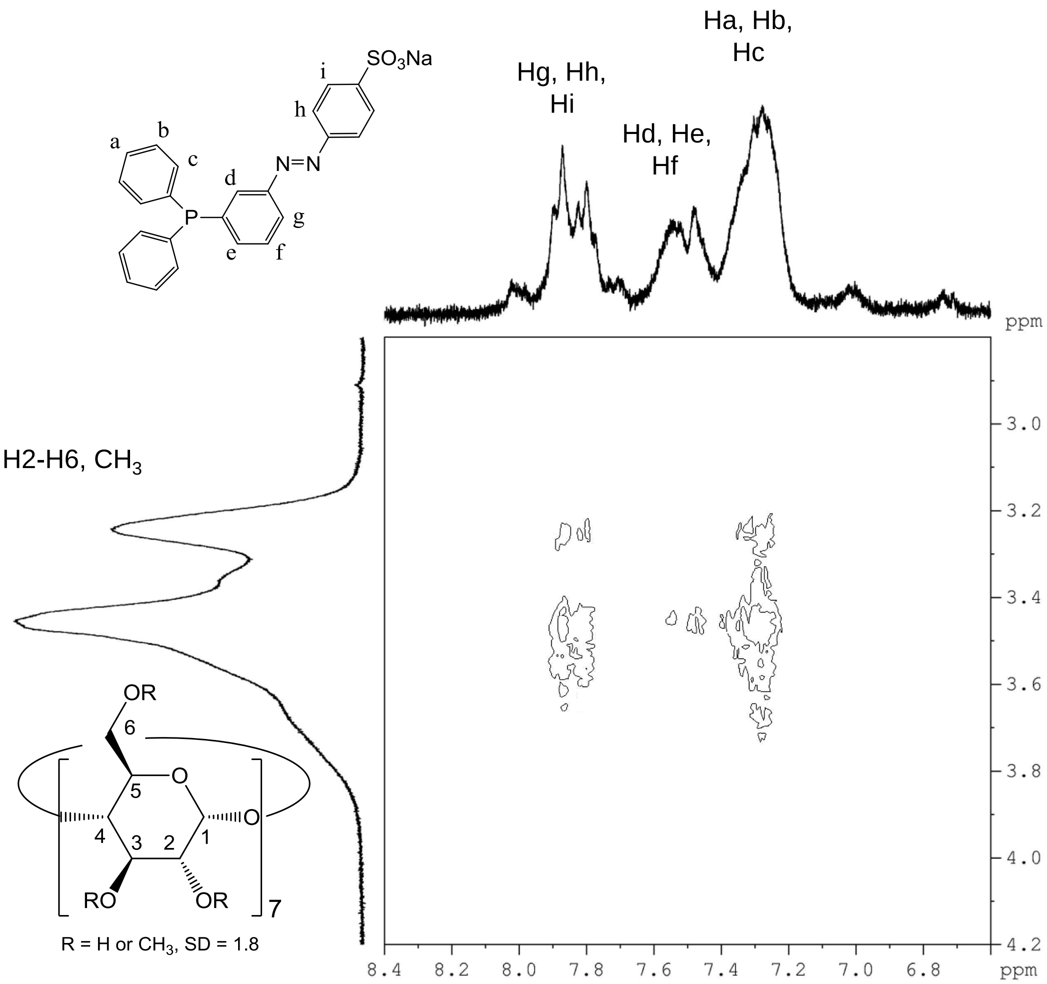

Figure S9: T-ROESY spectrum (300 MHz) of a stoichiometric mixture of RAME-β-CD and phosphane **2** (3 mM each) in D_2O at 20 °C.

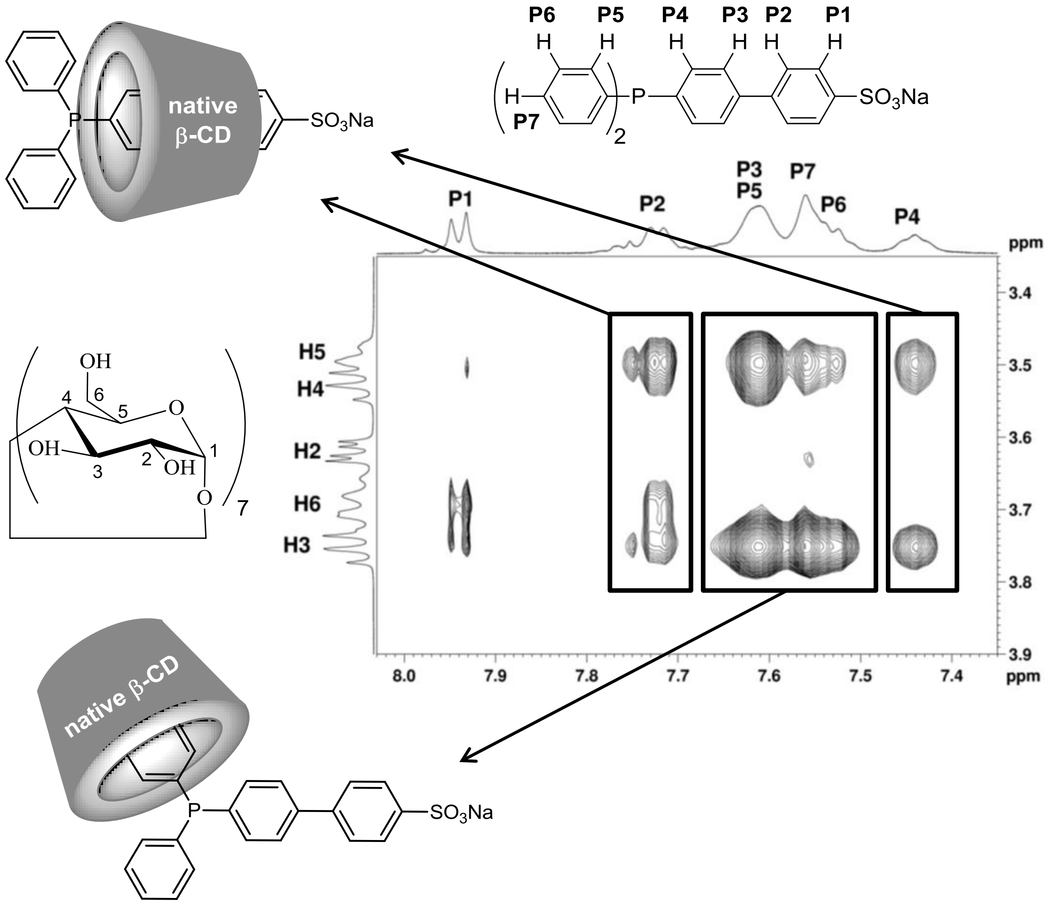

Figure S10. T-ROESY spectrum (500 MHz) of a stoichiometric mixture of native β-CD and phosphane **3** (10 mM each) in D_2O at 20 °C.

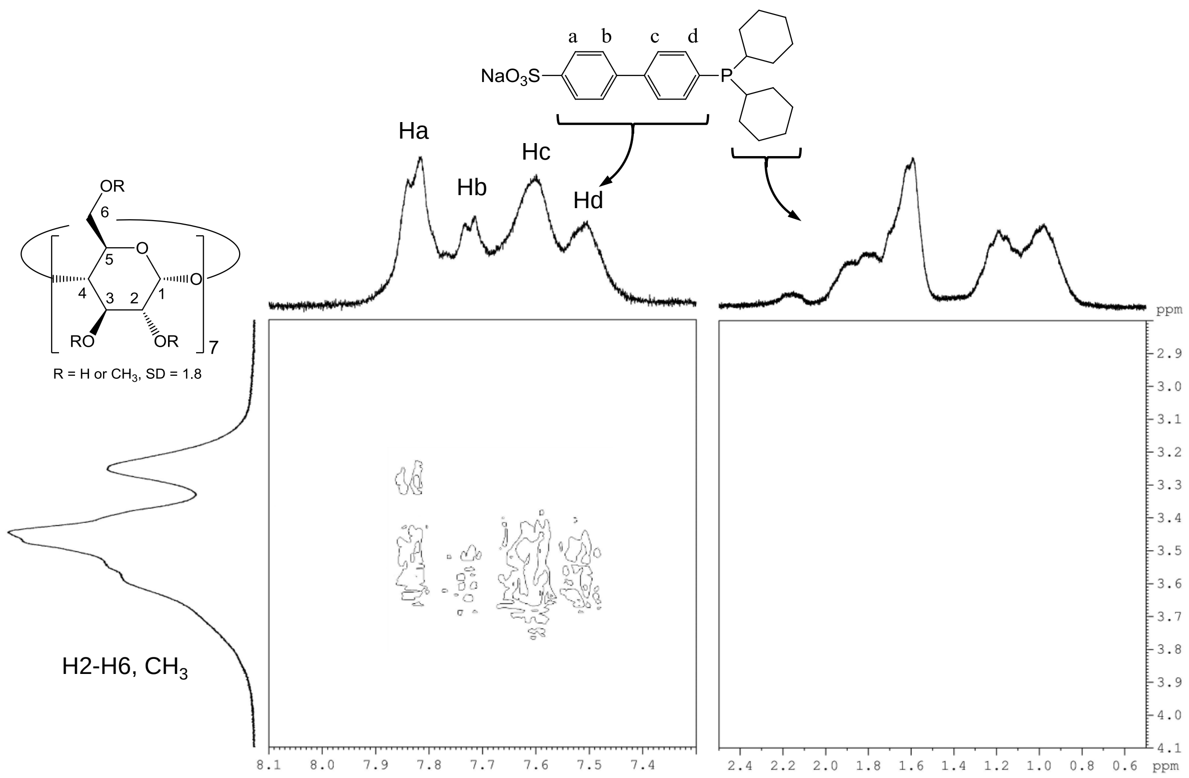

Figure S11: T-ROESY spectrum (300 MHz) of a stoichiometric mixture of RAME-β-CD and phosphane **4** (3 mM each) in D_2O at 20 °C.

Palladium-catalyzed Tsuji–Trost reaction

$Pd(OAc)_2$ (2.23 μmol, 0.5 mg) and phosphane (20.1 μmol, 9 equiv) were introduced under a nitrogen atmosphere into a Schlenk tube containing water (2 g). After stirring with a magnetic bar for 16 h, the yellow solution was transferred into a mixture of allyl undecyl carbonate (223 μmol, 100 equiv), diethylamine (446 μmol, 200 equiv), heptane (2 g), dodecane (110 μmol, 50 equiv) as internal standard and 20.1 μmol of RAME-β-CD (9 equiv/Pd; 1 equiv with respect to the phosphane for the best conditions). The medium was stirred at 1250 rpm at room temperature and the reaction was monitored by quantitative gas chromatographic analysis of the organic layer.
